# Altered Functional Brain Network in Systemic Lupus Erythematosus Patients Without Overt Neuropsychiatric Symptoms Based on Resting-State Functional Magnetic Resonance Imaging and Multivariate Pattern Analysis

**DOI:** 10.3389/fneur.2021.690979

**Published:** 2021-07-21

**Authors:** Yiling Wang, Muliang Jiang, Lixuan Huang, Xia Meng, Shu Li, Xiaoqi Pang, Zisan Zeng

**Affiliations:** Department of Radiology, The First Affiliated Hospital of Guangxi Medical University, Nanning, China

**Keywords:** systemic lupus erythematosus, resting-state functional MRI, degree centrality, functional connectivity, multivariate pattern analysis

## Abstract

**Objective:** This study aims to investigate the alterations in functional brain network in systemic lupus erythematosus patients without overt neuropsychiatric symptoms [neuropsychiatric systemic lupus erythematosus (non-NPSLE)] from the perspective of degree centrality (DC) and functional connectivity (FC) using resting-state functional magnetic resonance imaging (MRI) and multivariate pattern analysis (MVPA) approach.

**Methods:** DC analysis was performed based on the resting-state functional MRI data derived from 47 non-NPSLE patients and 47 healthy controls (HCs). Nodes with abnormal DC were utilized as seeds for further FC analysis. The correlation between MRI variables and clinical or neuropsychological data was analyzed using Pearson correlation analysis. Finally, MVPA classification based on DC was performed.

**Results:** When compared with the HCs, the non-NPSLE patients exhibited remarkably higher DC in the bilateral hippocampus (HIP), right insula (INS), and lower DC in the left superior parietal gyrus. Furthermore, the patients displayed significantly higher FC between the left HIP and the left INS/left dorsolateral middle frontal gyrus/left supramarginal gyrus and higher FC between the right HIP and the right middle temporal gyrus/right dorsolateral middle frontal gyrus/right dorsolateral inferior frontal gyrus/right supramarginal gyrus (all imaging variables mentioned earlier underwent cluster-level false discovery rate corrections, the voxel threshold was *p* < 0.001, cluster threshold was *p* < 0.05). Correlation analysis revealed significantly negative correlations between DC values of the right INS and disease activity and the DC values of the right HIP and the Montreal Cognitive Assessment scores. The accuracy, sensitivity, and specificity of MVPA classification based on DC were 72.34, 63.83, and 80.85%, respectively. The most discriminative power brain regions were chiefly located within the temporal, parietal, and frontal regions.

**Conclusion:** Patients with non-NPSLE exhibited abnormal DC and FC in the brain network. MVPA based on DC possessed commendable classification ability. Our study may provide a novel perspective on the neuropathological mechanisms underlying subclinical brain damage in non-NPSLE.

## Introduction

Systemic lupus erythematosus (SLE) is an autoimmune disease that affects multiple systems, which may affect the nervous system, progressing to neuropsychiatric systemic lupus erythematosus (NPSLE). NPSLE is prevalent and is associated with a high rate of disability and mortality (1). The American College of Rheumatology (ACR) has defined 19 neuropsychiatric symptoms in NPSLE patients (2). Among them, cognitive impairment is one of the most common symptoms, which significantly reduces the patients' quality of life (3) and manifests subtly in SLE patients without overt neuropsychiatric symptoms (defined as non-NPSLE) (4). Neuroimaging studies have also found evidence of gray matter atrophy (5, 6), microstructure damage of white matter (7), and abnormal neurochemical metabolism in non-NPSLE (8), suggesting that subclinical brain damage may occur before it develops into NPSLE. Therefore, identifying such brain damage and clarifying its mechanism would be clinically beneficial.

Resting-state functional magnetic resonance imaging (rs-fMRI) detects spontaneous neuronal activity in the resting state and is capable of discovering brain abnormalities that are invisible on structural MRI. It is considered to be a suitable approach to unravel the neural mechanisms of neuropsychiatric disorders (9). Several previous rs-fMRI studies have confirmed aberrant regional activities in patients with non-NPSLE (10, 11). However, the human brain is highly interconnected under physiological circumstances, and the brain function is managed by a spatially distributed network rather than by the spontaneous activities of local regions (12, 13). Investigation of the brain network is necessary for a comprehensive understanding of the brain alteration induced by disease. Brain network anomaly has been previously explored in non-NPSLE. Using the independent component analysis method, Nystedt et al. (14) found the increased functional connectivity (FC) within the default mode network (DMN) and central executive network and in-between these two sub-networks in non-NPSLE. Another study detected altered FC within the DMN, salient network, central executive network, and working-memory network (15). These findings were extremely useful in uncovering FC abnormalities within and between specific subnetworks but only provided limited information on the whole functional connectome. Recently, graph theory-based network analysis has been used to decipher the connections within the entire brain network (16). Degree centrality (DC) is one of the measurements of global connections derived from the graph theory. It is specifically used to expound any nodes' FC information by calculating the number of links between a node and the rest of the brain (17). DC is a reliable indicator with the advantages of high sensitivity and repeatability, and it has been successfully used as a neuroimaging biomarker to visualize network abnormalities in the early stages of many neuropsychiatric diseases (18, 19). So far, however, little is known about the DC alterations in non-NPSLE patients.

With the rise of machine learning in radiology, researchers have made lots of attempts to differentiate patients with neuropsychiatric diseases from the healthy population (20). Multivariate pattern analysis (MVPA), an emerging approach of MRI data machine learning, has been successfully used to find potential neuroimaging biomarkers helpful in individual diagnostic decisions (21). Furthermore, on the basis of distributed discriminative features, MVPA can also be used in an exploratory manner to further detect the prominently discriminative regions underlying the brain damage of disease (22, 23). Thus, the combined application of MVPA help in exploring the value of abnormal DC in the detection of subclinical brain injury in SLE and characterizing the distribution of brain regions with abnormal network centrality, and this aspect has not been studied so far.

In this study, the alteration in network centrality of each node was captured by DC comparison between non-NPSLE patients and HCs. Subsequently, we conducted a further seed-based FC analysis using brain regions with abnormal DC as seeds, aiming to reveal their significant abnormal connectivity with other regions. MVPA was used to explore the classification ability of machine learning based on the patterns of DC. We also investigated the possible associations between brain functional alterations and clinical variables or neuropsychological performance.

## Materials and Methods

### Participants

The SLE patients were selected from the in- and outpatients of the Department of Rheumatism and Immunology in the First Affiliated Hospital of Guangxi Medical University in Nanning, China. Also, the age-, sex-, and education-level matched healthy controls were recruited from the general Nanning population between May 2019 and October 2020. Two experienced rheumatology immunologists were responsible for the diagnoses of SLE patients by the standard of the ACR classification criteria for SLE (24). Both SLE patients and HCs met the following inclusion criteria: (1) age range: 15–50 years; (2) right-handed; and (3) able to cooperate with MRI data acquisition and neuropsychological assessment.

The following SLE patients were excluded from the study: (1) patients with ACR-established NPSLE symptoms (2); (2) those suffering from other autoimmune diseases; (3) those with a neurological or psychiatric disease history, such as Alzheimer's disease and depressive disorder; (4) those with intracranial damage such as tumor, brain trauma, and history of brain surgery; (5) those displaying any high white matter intensity on T2-fluid-attenuated inversion recovery sequences or cerebral atrophy; (6) those suffering from diabetes, hypertension, and other chronic diseases that may affect brain function; (7) those suffering from mild or more severe depression [Beck Depression Inventory II (BDI-II) score > 13] (25); (8) those abusing alcohol or drugs; (9) those who were pregnant or were planning to get pregnant; and (10) those with contraindications to MRI assessment, for example, fear of closed spaces.

We excluded HCs with psychiatric/neurological diseases, diseases affecting brain function (e.g., diabetes, hyperthyroidism, etc.), structural abnormalities in the brain, and drug or alcohol abuse.

This study received approval from the Medical Ethics Committee of the First Affiliated Hospital of Guangxi Medical University. Before commencing the research, all participants were fully informed of the purpose, processes, and matters needing attention of the study and volunteered to give consent for participation.

### Clinical Data

For the patient group, we used standardized methods to record the following clinical information of the subjects from their medical documents: disease duration, onset age, laboratory serological indexes (C3, C4, anti-dsDNA antibody, and erythrocyte sedimentation rate), and disease activity measured using the Systemic Lupus Erythematosus Disease Activity Index (SLEDAI). All data were acquired on the day of the MRI examination.

### Neuropsychological Tests

The Montreal Cognitive Assessment (MoCA) was applied to evaluate the subject's cognitive performance, and a low score was indicative of cognitive decline (26). To measure the depression status, the BDI-II (25, 27) was used, and a high score represented a poor mood state. The neuropsychological tests mentioned earlier were completed before the MRI assessment.

### Magnetic Resonance Imaging Data Acquisition

The MRI scan was conducted using a 3.0-T MR scanner (Siemens, Prisma) and a 64-channel head coil at the First Affiliated Hospital of Guangxi Medical University. Noise influence was eliminated with earplugs, and head movement was restricted with cushions. Initially, conventional MRI scans, including T1- and T2-weighted and T2-fluid-attenuated inversion recovery sequences, were performed on each participant to exclude the subjects with overt abnormal findings. In the subsequent formal study, the participants were directed to close their eyes but remain awake, prevent thinking, and refrain from head movements. For each subject, T1-weighted anatomical images were collected using a volumetric three-dimensional magnetization prepared by a rapid-acquisition gradient-echo sequence with the following parameters: repetition time = 2,200 ms, echo time = 2.48 ms, flip angle = 8°, slices = 172, slice thickness = 1 mm, field-of-view = 230 × 230 mm, and voxel size = 0.9 × 0.9 × 1 mm^3^. The rs-fMRIs were recorded by using an echo-planar imaging sequence (repetition time = 2,000 ms, echo time = 30 ms, flip angle = 90°, field-of-view = 192 × 192 mm^2^, slices = 37, thickness = 4 mm, and voxel size = 3 × 3 × 4 mm^3^); 240 volumes were transversely acquired. An experienced radiologist assisted in the data acquisition.

### Data Preprocessing

The images were preprocessed using RESTplusV1.2 (http://www.restfmri.net) and SPM12 (https://www.fil.ion.ucl.ac.uk/spm/) on MATLAB 2013b. First, the DICOM files were changed to the NifTI format. Further steps are as listed below: (1) The first 10 volumes were removed to attain stable magnetization. (2) The time difference between the slices was corrected. (3) Head motion was corrected with realignment in all subjects, and any subjects with head motion > 2.5 mm translation and 2.5° rotation in any direction were excluded from the statistical analysis. (4) The T1-weighted images were co-registered with the mean functional images and then segmented. (5) The functional image was standardized into the Montreal Neurological Institute space using the same standardization parameters as the structural image, and the generated image was resampled with a resolution of 3 × 3 × 3 mm3. (6) Linear detrend, nuisance covariate regression (including six head motion parameters and the signals of white matter and cerebrospinal fluid), and band-pass filtering (0.01–0.08 Hz) were done. (7) Finally, all images were smoothed using a 6-mm full-width at half maximum Gaussian kernel.

### Resting-State Functional Magnetic Resonance Imaging Data Calculations

Both DC and FC maps were analyzed using the RESTplus V1.2 based on the preprocessed data (rs-fMRI data used for DC calculation were not smoothed during preprocessing). Voxel-wise DC calculations were performed based on a threshold of *r* > 0.25, consistent with studies by other researchers (28, 29). Later, the global gray matter mean DC was subtracted from the resultant voxel-wise DC maps and then divided by the standard deviation of the gray matter DC to produce a *z* score map. Subsequently, the binary DC *z* score maps were spatially smoothed with a 6-mm full-width at half-maximum Gaussian kernel to reduce spatial noise.

Regions with abnormal DC *via* group comparison were utilized as seed for subsequent seed-based FC analysis in a seed-to-voxel manner to explore the detailed information regarding the connectivity in these regions. The reference time series for each seed was calculated by averaging the time series of the voxels within the seeds. Next, the time series of each voxel in the gray matter was obtained. The correlation coefficients between the time course from each seed and that from all voxels within the gray matter were calculated. Finally, Fisher's *r*-to-*z* transformation was used to normalize the correlation coefficients.

### Multivariate Pattern Analysis Approach

To assess the accuracy of differentiating the non-NPSLE patients from the HCs based on the DC value, MVPA was performed on the MATLAB platform using the pattern recognition for neuroimaging (PRoNTo) baggage. The following steps were involved in this approach: First, the functional image of each subject was projected as a point on the high-dimensional space outlined by the DC maps. Then, the individual brain maps were divided by a linear decision boundary, which was defined by the binary support vector machine classifier according to the preset category label. Hyperplane optimization was subsequently performed. The classifier performance was verified using the leave-one-out cross-validation method. The notion of this method was to remove one subject from each group to test the classifier trained by the rest of the subjects. Next, three indicators (accuracy, specificity, and sensitivity of DC classification) were extracted to evaluate the performance of MVPA. A non-parametric test was then conducted to estimate the statistical significance of the indicators mentioned earlier, and such a non-parametric test repeated the classification step 1,000 times. Finally, the weights of the brain regions were calculated to assess the importance of brain areas in classification. The voxels with the top 20% weight coefficient were summarized in the result (30).

### Statistical Analysis

All numeric data, including demographics, clinical data, and neuropsychological test scores, were compared using SPSS software, version 24.0. First, the Kolmogorov–Smirnov test was utilized to evaluate whether the data followed a normal distribution. For the normally distributed variables, a two-sample *t*-test was applied. Non-normally distributed data were analyzed by parametric test, and the sex data were tested by the chi-square test. *P* < 0.05 was considered statistically significant.

Two sample *t*-test was used to compare the group differences in DC and FC by using age, sex, and education as the covariates. The cluster-level false discovery rate (FDR) corrections (voxel threshold, *p* < 0.001; cluster threshold, *p* < 0.05) were used for multiple correction of DC and FC results.

The DC and FC values of the altered brain regions were extracted by utilizing RESTplus for the patient group. Then, Pearson correlation analyses were conducted in SPSS 24.0 to investigate the correlation between the DC or FC values and clinical data or neuropsychological test scores. The significance level was set to *p* < 0.05.

## Results

### Demographic, Clinical, and Neuropsychological Characteristics

After strict selection, we included 47 non-NPSLE patients and 47 HCs in this study. The non-NPSLE patients and HCs had statistically similar age, sex, and education (*p* > 0.05). Compared with the HCs, the non-NPSLE patients showed significantly poor performance with lower scores in the MoCA (*p* = 0.000 < 0.05), suggesting the patients group had a mild decline in cognition but did not meet the diagnostic criteria of cognitive impairment (MoCA score < 26). Also, the non-NPSLE patients showed worse performance on tests of depression with higher scores in BDI-II (*p* = 0.000 < 0.05), which indicated the patients had minimal mood disorders. The details are summarized in Table 1.

**Table 1:** Demographics, clinical data, and neuropsychological test scores of non-NPSLE patients and healthy controls.

	**Non-NPSLE patients (*n* = 47)**	**Healthy controls (*n* = 47)**	***t*-value**	***p*-value**
Gender (male/female)	9/38	11/36	N/A	0.80^a^
Age (years)	29.17 ± 9.23	27.02 ± 5.25	1.39	0.17^b^
Education (years)	12.32 ± 2.62	12.98 ± 2.49	−2.81	0.21^b^
Disease duration (months)	44.53 ± 56.39	N/A	N/A	N/A
Onset age (years)	27.72 ± 10.76	N/A	N/A	N/A
SLEDAI (scores)	13.23 ± 7.12	N/A	N/A	N/A
C3 (g/L)	0.67 ± 0.39	N/A	N/A	N/A
C4 (g/L)	0.16 ± 0.16	N/A	N/A	N/A
Anti-dsDNA ab (IU/l)	113.13 ± 115.31	N/A	N/A	N/A
ESR (mm)	45.60 ± 26.77	N/A	N/A	N/A
MoCA (scores)	26.72 ± 1.25	28.49 ± 1.14	−1.76	<0.001^b^^*^
BDI-II (scores)	10.04 ± 2.78	6.40 ± 2.10	9.77	<0.001^b^^*^

*Data are shown as mean ± SD unless otherwise indicated*.

a *Chi-square test*.

b *Two-sample t-tests*.

* *The results were statistically significant (p < 0.05)*.

*Non-NPSLE, non-neuropsychiatric systemic lupus erythematosus; SLEDAI, Systemic Lupus Erythematosus Disease Activity Index; Anti-dsDNA ab, anti-dsDNA antibody; ESR, erythrocyte sedimentation rate; MoCA, Montreal Cognitive Assessment; BDI-II, Beck Depression Inventory II; N/A, not applicable*.

### Group Difference in Degree Centrality

**Figure 1:**
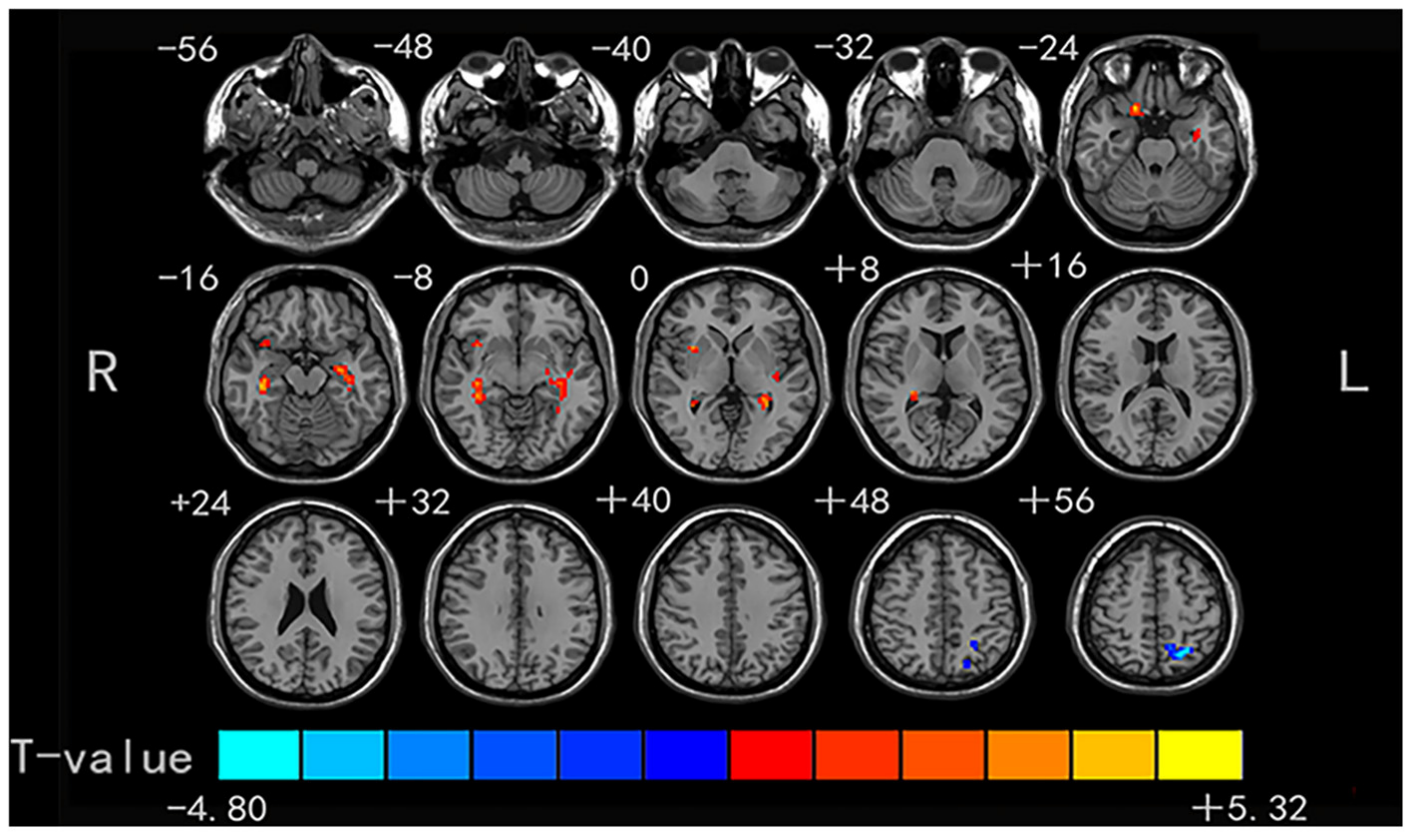
Comparison of DC values between non-NPSLE patients and healthy controls. Warm and cold colors' areas represent brain regions where DC values of non-NPSLE patients were significantly higher and lower than those of healthy controls, respectively. Cluster-level FDR correction with a voxel threshold of *p* < 0.001 and cluster threshold of *p* < 0.05.

Compared with the HCs, the non-NPSLE patients displayed significantly higher DC values in the right insula (INS) and bilateral hippocampus (HIP) and lower DC values in the left superior parietal gyrus (SPG) (cluster-level FDR corrected; the voxel threshold was *p* < 0.001, the cluster threshold was *p* < 0.05, and the cluster size was >70). Details of the brain areas with aberrant DC values are presented in Table 2 and Figures 1, **3**.

**Table 2:** DC analysis results.

**Brain region**	**Cluster size**	***t*-value**	**Peak MNI coordinates (X, Y, Z)**
**Increased DC in non-NPSLE patients**			
INS.R	75	4.66	(18, 18, −24)
HIP.L	181	5.32	(−30, −12, −18)
HIP.R	106	5.33	(36, −24, −12)
**Decreased DC in non-NPSLE patients**			
SPG.L	104	−4.77	(−24, −60, 57)

*MNI, Montreal Neurological Institute; Non-NPSLE, Non-neuropsychiatric systemic lupus erythematosus; DC, Degree centrality; INS.R, Right insula; HIP.L, Left hippocampus; HIP.R, Right hippocampus; SPG.L, Left superior parietal gyrus*.

*Cluster-level FDR correction with a voxel threshold of p < 0.001 and cluster threshold of p < 0.05*.

### Group Difference in Functional Connectivity

**Figure 2:**
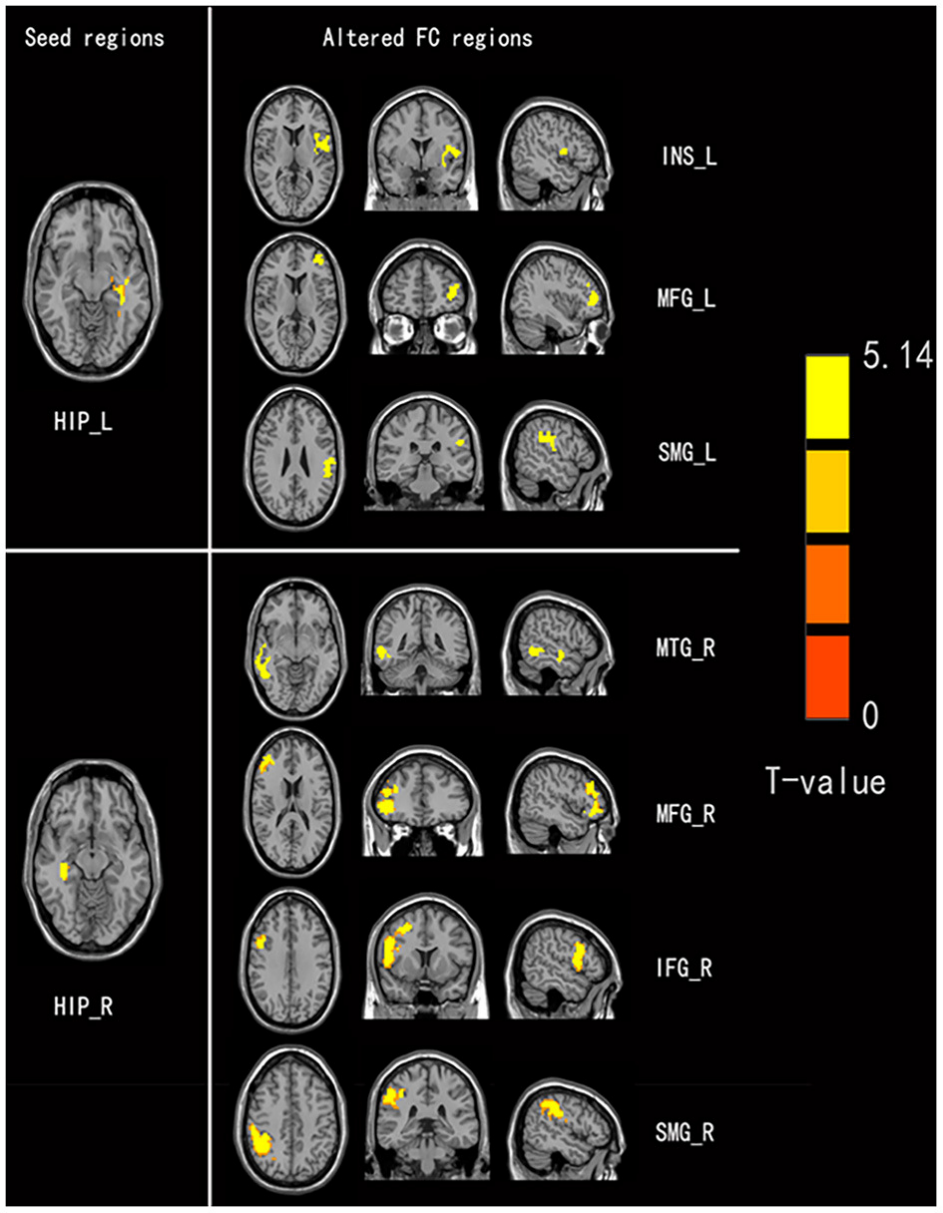
Comparison of seed-based FC values between non-NPSLE patients and healthy controls (seed are left hippocampus and right hippocampus, respectively). Warm color's regions represent brain areas with significantly increased FC values in non-NPSLE patients *vs*. that in healthy controls. Cluster-level FDR correction with a voxel threshold of *p* < 0.001 and cluster threshold of *p* < 0.05 (FC, Functional connectivity; HIP.L, Left hippocampus; INS.L, Left insula; MFGdor.L, Left dorsolateral middle frontal gyrus; SMG.L, Left supramarginal gyrus; HIP.R, Right hippocampus; MTG.R, Right middle temporal gyrus; MFGdor.R, Right dorsolateral middle frontal gyrus; IFGdor.R, Right dorsolateral inferior frontal gyrus; SMG.R, Right supramarginal gyrus).

**Figure 3:**
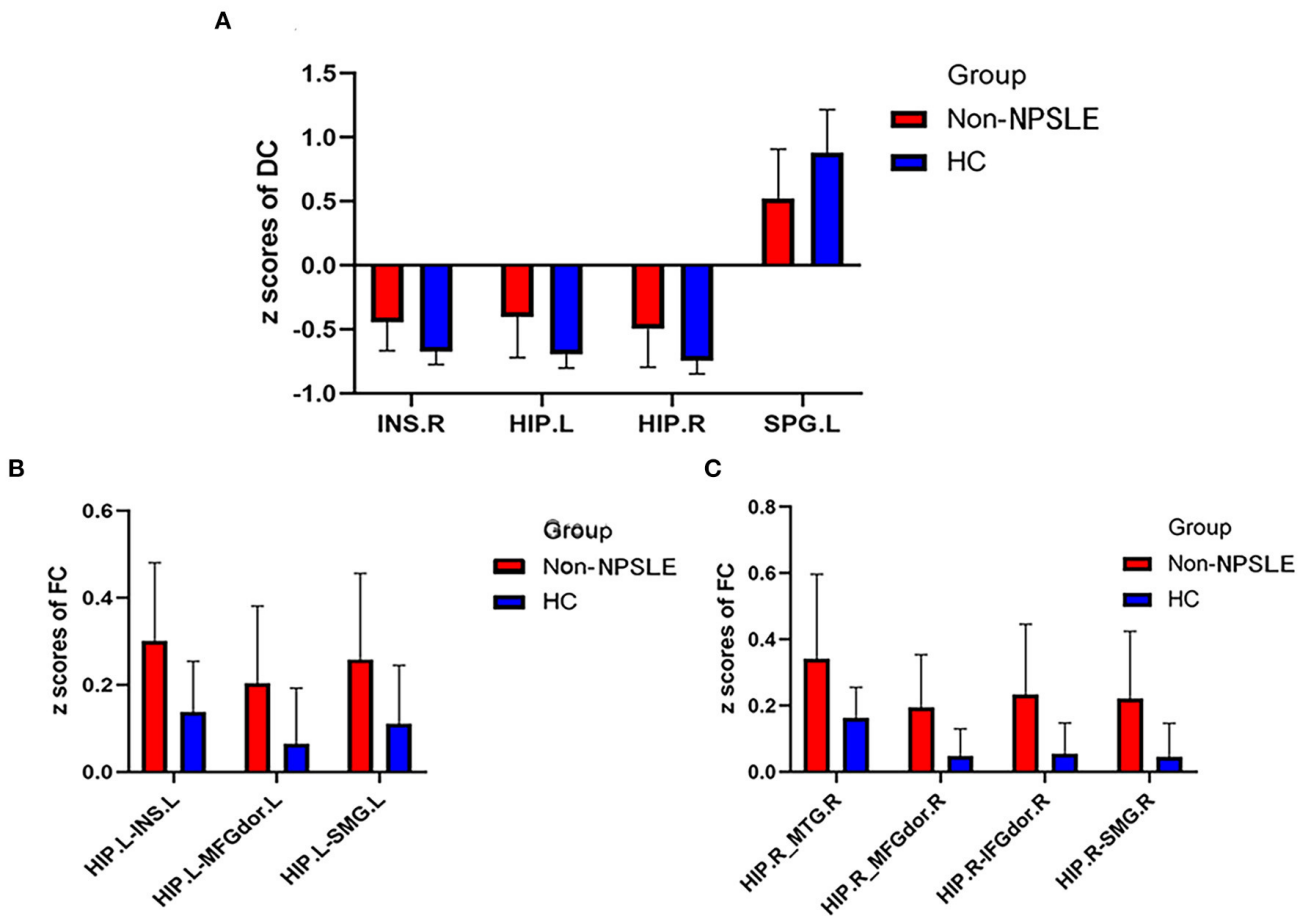
Comparison of DC *z* scores and FC *z* scores between two groups (DC, Degree centrality; FC, Functional connectivity; Non-NPSLE, Non-neuropsychiatric systemic lupus erythematosus; HC, Healthy control; INS.R, Right insula; HIP.L, Left hippocampus; HIP.R, Right hippocampus; SPG. L, Left superior parietal gyrus; INS.L, Left insula; MFGdor.L, Left dorsolateral middle frontal gyrus; SMG.L, Left supramarginal gyrus; MTG.R, Right middle temporal gyrus; MFGdor.R, Right dorsolateral middle frontal gyrus; IFGdor.R, Right dorsolateral inferior frontal gyrus; SMG.R, Right supramarginal gyrus). **(A)** DC *z* scores of non-NPSLE vs. HC. **(B)** FC *z* scores of non-NPSLE vs. HC. **(C)** FC *z* scores of non-NPSLE vs. HC.

Four brain regions (right INS, bilateral HIP, and left SPG) that showed significant differences in DC between non-NPSLE patients and HCs were set as seed. We further conducted seed-based FC between these seeds and the whole brain regions. When compared with the HCs, the non-NPSLE patients exhibited higher FC values between the left HIP and left INS/left dorsolateral middle frontal gyrus/left supramarginal gyrus (SMG) and higher FC values between the right HIP and right middle temporal gyrus/right dorsolateral middle frontal gyrus/right dorsolateral inferior frontal gyrus/right SMG (cluster-level FDR corrected; the voxel threshold was *p* < 0.001, the cluster threshold was *p* < 0.05, and the cluster size was >172). However, when the bilateral hippocampus was set as seed, no decreased FC between these two seeds and other brain regions was found in this study. In addition, no altered FC was discovered when the right INS or left SPG was used as the seed. Details of the brain regions with abnormal FC are summarized in Table 3 and Figures 2, 3.

**Table 3:** FC analysis results.

**Seed region**	**Increased connected region**	**Cluster size**	***t*-value**	**Peak MNI coordinates (X, Y, Z)**
HIP.L	INS.L	273	4.66	(−54, −3, 6)
	MFGdor.L	173	4.37	(−36, 45, 3)
	SMG.L	172	4.25	(−66, −21, 21)
HIP.R	MTG.R	270	4.26	(57, −51, −9)
	MFGdor.R	368	5.06	(48, 33, 30)
	IFGdor.R	477	5.16	(54, 6, 9)
	SMG.R	806	5.41	(39, −48, 42)

*MNI, Montreal Neurological Institute; HIP.L, Left hippocampus; INS.L, Left insula; MFGdor.L, Left dorsolateral middle frontal gyrus; SMG.L, Left supramarginal gyrus; HIP.R, Right hippocampus; MTG.R, Right middle temporal gyrus; MFGdor.R, Right dorsolateral middle frontal gyrus; IFGdor.R, Right dorsolateral inferior frontal gyrus; SMG.R, Right supramarginal gyrus*.

*Cluster-level FDR correction with a voxel threshold of p < 0.001 and cluster threshold of p < 0.05*.

### Correlation Analysis

**Figure 4:**
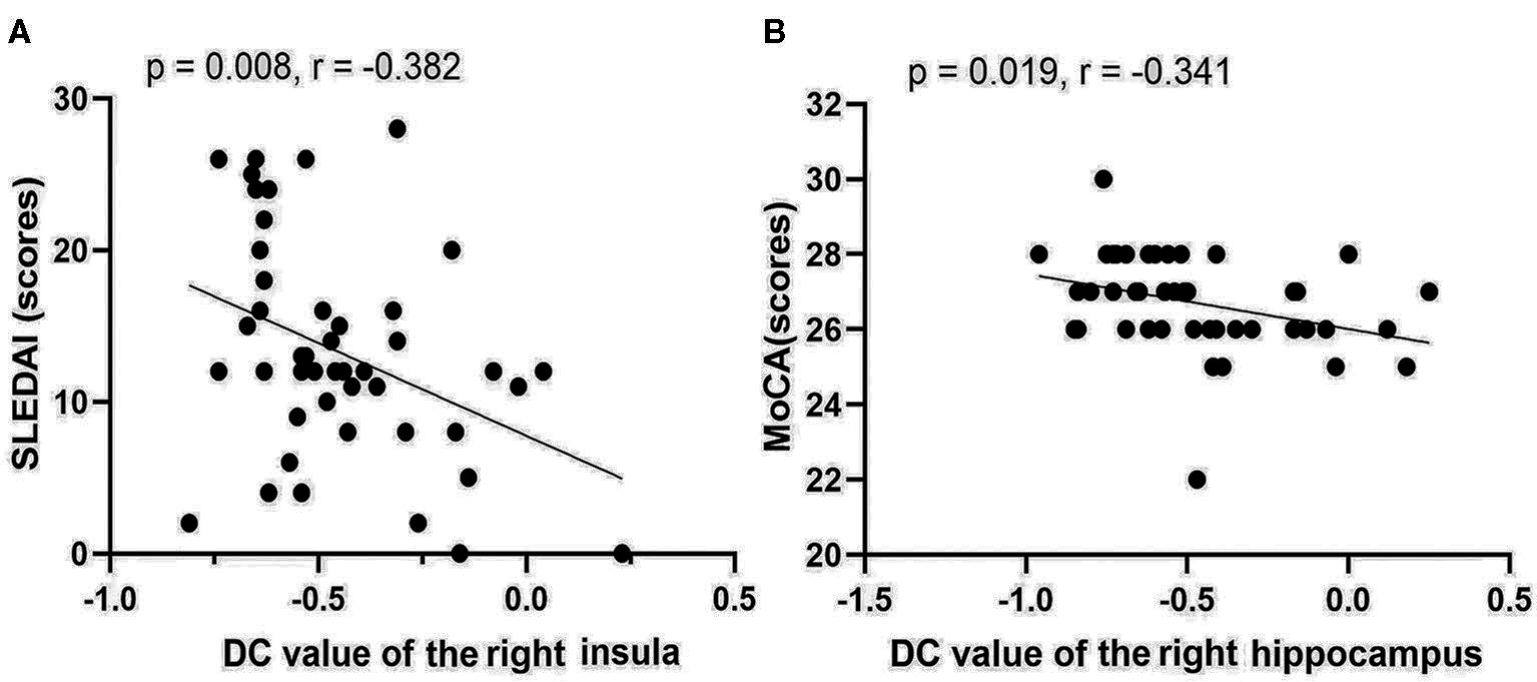
Correlations among rs-fMRI data, clinical data, and neuropsychological test scores of non-NPSLE patients. **(A)** DC value of right insula vs. SLEDAI (scores). **(B)** DC value of right hippocampus vs. MoCA (scores) (SLEDAI, Systemic Lupus Erythematosus Disease Activity Index; MoCA, Montreal Cognitive Assessment).

For the non-NPSLE patients, the DC values of the right INS were negatively correlated with the SLEDAI scores (*r* = −0.382, *p* = 0.008). A negative correlation was found between the DC values of the right HIP and the MoCA scores (*r* = −0.341, *p* = 0.019). However, no significant correlations were observed between the aberrant FC values in the brain regions and the clinical data or the neuropsychological test scores (*p* > 0.05). Details of the correlation analysis are presented in Figure 4.

### Multivariate Pattern Analysis Results

**Figure 5:**
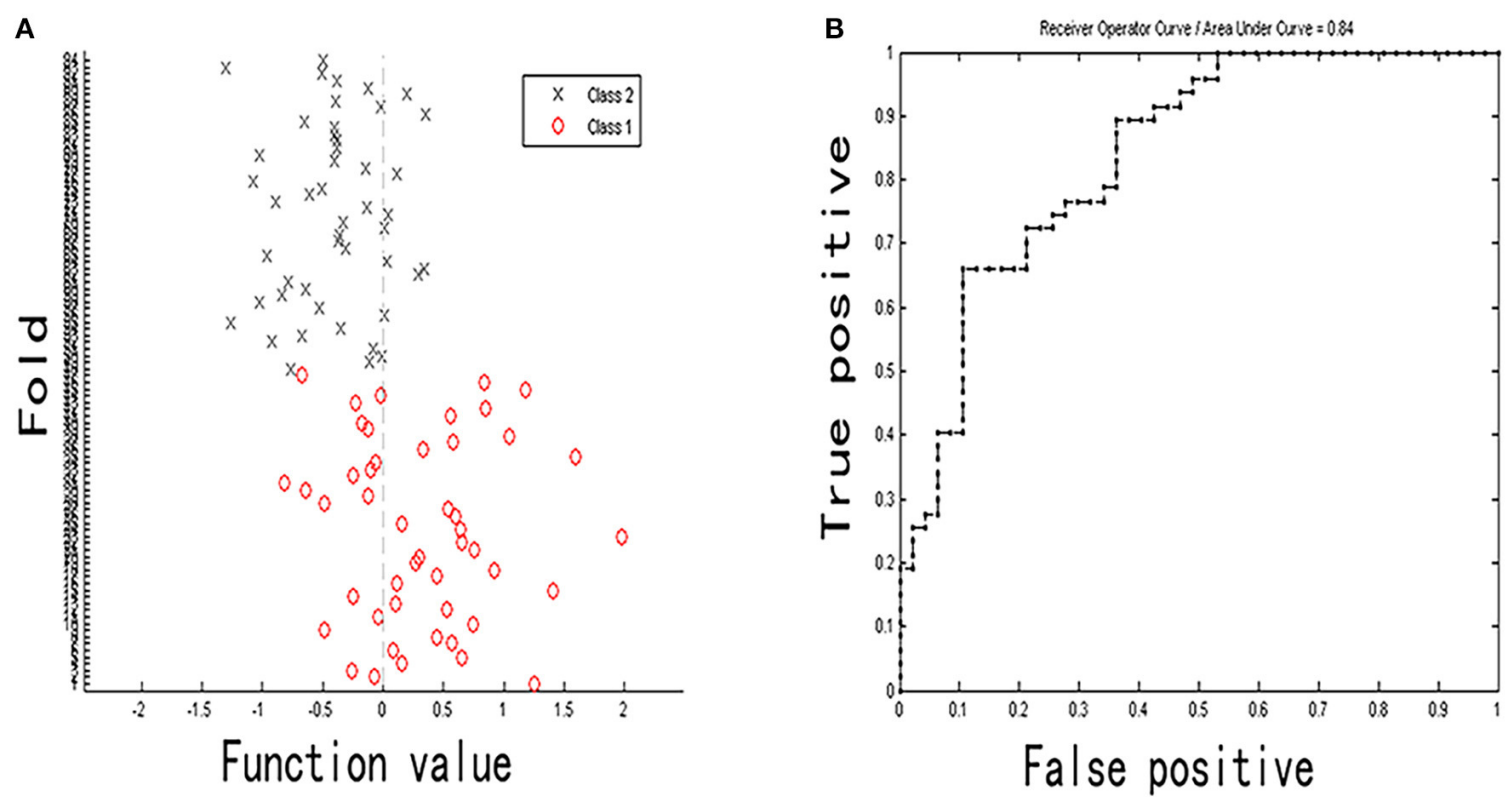
Classification plot **(A)** and area under receiver operating characteristic curve **(B)** for non-NPSLE patients vs. controls classification based on DC values (class 1 is patient group and class 2 is control group).

**Figure 6:**
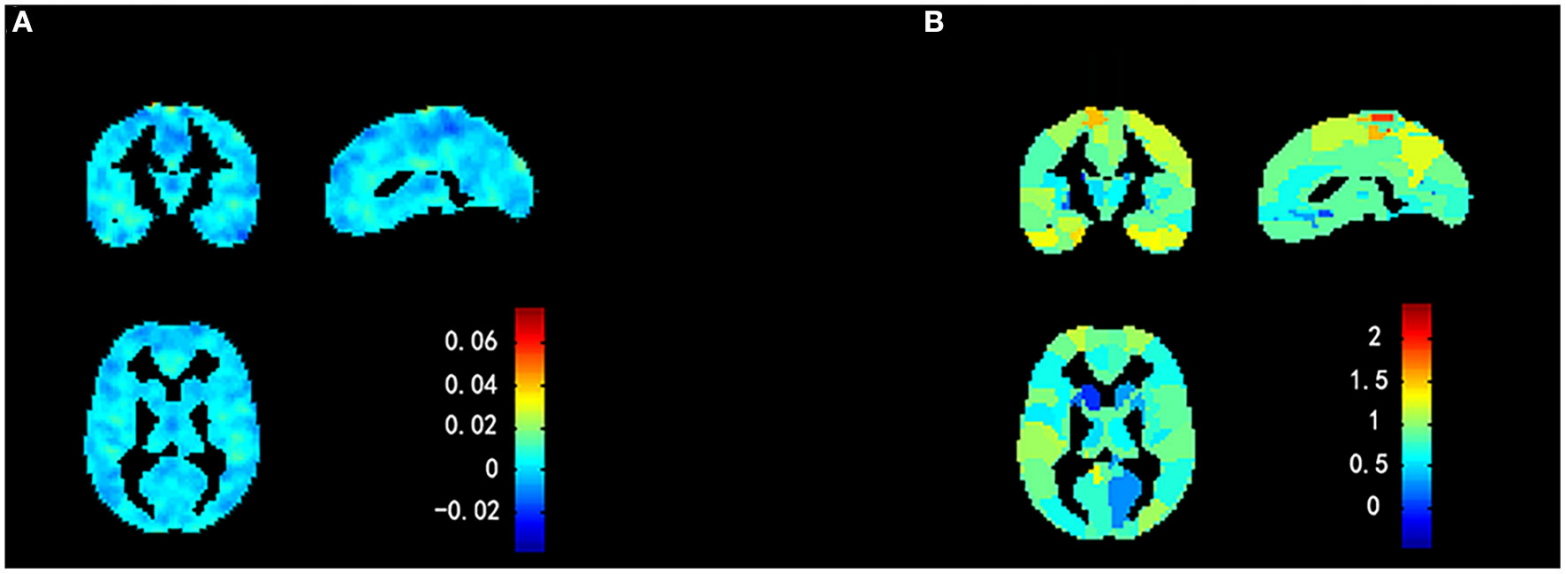
Weight maps for classification model using a 2-fold cross-validation framework on whole sample. **(A)** Voxel-based predictive pattern. Color bar indicates weight of voxels for classification. **(B)** Region-based pattern localization map calculated from voxel-based predictive pattern showed in **(A)**. Color bar uncovers percent of weights that each anatomical region represents.

The classification (based on DC values) accuracy was 72.34%, and the sensitivity and specificity were 63.83 and 80.85%, respectively (*p* < 0.05). The area under the receiver operating characteristic curve values of the classification model was 0.84. The most valuable discriminative brain regions with top 20% weight coefficient in the classification overlapped in the temporal, parietal, and frontal regions (the order from high to low in weight was left inferior temporal gyrus, right middle temporal gyrus, right superior parietal gyrus, right paracentral lobule, right parahippocampus, right inferior occipital gyrus, and left inferior parietal gyrus). Details of the MVPA results are presented in Figures 5, 6.

## Discussion

Using the combination of DC and seed-FC analyzes, we investigated the nodes' FC within the brain network in patients with non-NPSLE. Also, the application of MVPA may help explore the feasibility of using DC as a discriminant feature of non-NPSLE subclinical brain injury. Our findings indicate the following: (a) when compared with the HCs, the non-NPSLE patients exhibited increased DC in bilateral HIP and right INS and decreased DC in left SPG. In addition, the patient group displayed enhanced FC between the bilateral HIP and the other brain regions, as described in Table 3, and there were no other abnormalities in the FC analysis; (b) patients with non-NPSLE showed a mild cognitive deficit, which may be associated with abnormal DC in the right HIP; (c) high disease activity in the non-NPSLE patients was related to abnormality in the brain network; and (d) MVPA based on DC pattern had good performance in differentiating the patients from the HCs, and the most discriminative power brain regions were mainly located within the temporal, parietal, and frontal regions, of which the left inferior temporal gyrus (ITG) was identified with the highest discriminative power. These findings provided us with a novel insight into the altered characteristics of brain functional network in the non-NPSLE patients.

In this study, we uncovered that the non-NPSLE patients displayed increased DC in bilateral HIP and right INS. Our findings were in accordance with a previous study, which applied DC methods and noted increased DC in the bilateral HIP (31). A region with high DC is defined as a network “hub,” which has increased importance within the brain network, and the network hubs play important roles in information integration (32). Both the insula and hippocampus have been shown to be closely connected to a wide network of cortical and subcortical brain regions and receive a large amount of information input from the cortex (33–35). This may be the anatomical basis for the increased number of functional connections between the hippocampus, insula, and other nodes. However, a previous topology analysis has demonstrated that nodes with high DC required more blood flow and increased metabolism, making them vulnerable to diseases (36). It could be speculated that a region with high DC is a target for disease and that its function is diminishing. The HIP is a core component of DMN (37), playing a role in diversified aspects of cognition, including episodic memories, attention, and perception (35). As such, we hypothesized the increased DC in HIP might be involved in the neuropathological mechanism of cognitive deficits in the non-NPSLE patients. The current study synergistically found that the higher DC values of the right HIP were correlated with worse cognitive performance (i.e., lower MoCA score), further supporting our hypothesis. Besides, it has been stated that increased blood flow and metabolism in the INS triggers the processing of internal sensory signals that are associated with arousal of anxiety and depression symptoms (38, 39). Our study established that the patient group displayed minimal mood disorders. Previous surveys (40) have shown that the prevalence of anxiety and depression in the SLE patients is twice as that in the normal population. Collectively, these findings suggest that increased DC in INS may be the neuropathological cause of mood disorders in SLE patients.

Conversely, we noted a reduction of DC in the left SPG, which agrees with a previous finding (31). Decreased DC implies a functional malfunction in the integration of nodes in a network (i.e., decompensation or unbalance) (41). The SPG is known as the terminal of the dorsal visual signal path and is involved in the process of visuospatial perception (42). A meta-analysis has reported that the non-NPSLE patients had deficits in visual attention, reasoning, and memory (4). As such, reduced DC in SPG may be the possible neural correlate of visual dysfunction in these patients.

The following seed-to-voxel FC analysis detected increased FC between the bilateral HIP and the other regions of the brain, as outlined earlier. Intriguingly, these findings seem to show a pattern of increased ipsilateral connections. It is speculated that there may be laterality of functional connection in patients with non-NPSLE. SMG is a portion of the inferior parietal lobe (37), whereas the dorsolateral MFG and IFG mainly belong to the dorsolateral prefrontal cortex (43). Hyperconnectivity was found between the hippocampus with the inferior parietal lobe, INS, and dorsolateral prefrontal cortex in this study, which parallels the previous research (14). Integrated research on non-NPSLE patients utilized diffusion tensor imaging, and fluorine-18 fluorodeoxyglucose positron emission tomography approaches found that the patients had compromised microstructural integrity in clusters that contained the HIP, frontal and parietal lobe (44). Remarkably, they also found that the areas with microstructural damage were adjacent to the gray matter with hypermetabolism, which was postulated as a compensatory neuronal mechanism. Hence, we hypothesize that the hyperconnectivity between these regions may also be a compensatory neuronal response to the disease-induced structural damage. Nevertheless, such a hypothesis needs to be confirmed by multimodal MRI studies combining structural and functional imaging in the future. Previous task-fMRI studies have shown that SLE patients had greater activation in the PFC, HIP, INS, and parietal lobe while performing certain cognitive tasks (45–47). These findings suggest that activations of these areas are involved in maintaining the cognitive functions in the SLE patients. Therefore, the hyperconnectivity between these regions may also serve as a compensatory mechanism for normal cognitive function.

The present study is the first-ever machine learning research based on the pattern of resting-state brain function in non-NPSLE patients. The results have shown that MVPA based on DC values is capable of distinguishing non-NPSLE patients from HCs, indicating that DC could serve as an objective discriminative feature in detecting subclinical brain damage in SLE at the individual level. Furthermore, this finding further confirms that abnormalities in the brain functional network of SLE patients precede the appearance of overt neuropsychiatric symptoms. The most discriminating regions with a high weight of classification were mainly distributed in the temporal, parietal, and frontal regions, unveiling the characteristics of DC alteration in non-NPSLE. Among these regions, the ITG possesses the highest discriminative power. Zhang et al. and Yu et al. consistently found aberrant local spontaneous brain activity in non-NPSLE patients using the amplitude of low-frequency fluctuation index. These findings may suggest that ITG is a specific target for the brain dysfunction of non-NPSLE patients.

Another striking finding of the current study is the negative correlation between the DC values in the right INS and the SLEDAI (higher the SLEDAI, higher is the disease activity of SLE), suggesting that high disease activity is related to low DC. In line with our finding, a previous study has confirmed that SLE patients with high disease activity had reduced N-acetyl aspartate/creatinine, signifying neuron injury (8). Taken together, we speculate that the high disease activity might result in malfunction and irreversible damage to the INS, thereby highlighting the clinical significance of disease activity control.

Nevertheless, our study has some limitations. First, although our findings showed altered FC in regions associated with cognitive and emotional processes, few significant correlations were found between DC/FC abnormalities and performance in the neuropsychological test. Thus, more research is needed to further prove the correlation. Second, this study lacks significant correlations between clinical data and rs-fMRI abnormalities, which may be caused by the insufficient sample size. Therefore, in future research, we need to expand the sample size to further explore the clinical relevance of these fMRI variables.

## Conclusion

To sum up, by combining DC and seed-based FC approaches, we detected the aberrant nodes with network DC and the corresponding altered connections with some other regions in the non-NPSLE patients. These findings indicate that brain networks could be damaged before the onset of overt neuropsychiatric symptoms. Also, we also noticed that these patients' cognitive deficits were correlated with the abnormal DC in the hippocampus. Furthermore, the MVPA results suggested that the rs-fMRI could help in identifying early brain damage in SLE patients. Disease activity may be a predictive clinical factor of brain dysfunction in non-NPSLE patients.

## Data Availability Statement

The original contributions presented in the study are included in the article/supplementary material, further inquiries can be directed to the corresponding author/s.

## Ethics Statement

The study was approved by the Ethics Committee of the First Affiliated Hospital of Guangxi Medical University. The written informed consent was obtained from all individual participants included in this study.

## Author Contributions

ZZ designed the study. YW, SL, and XM acquired the data. LH and XP performed the data analysis. XP and MJ interpreted the results. YW and MJ prepared the manuscript. All the authors contributed to manuscript revision and approved the final version for publication.

## Conflict of Interest

The authors declare that the research was conducted in the absence of any commercial or financial relationships that could be construed as a potential conflict of interest.
